# COVID stress syndrome: Concept, structure, and correlates

**DOI:** 10.1002/da.23071

**Published:** 2020-07-05

**Authors:** Steven Taylor, Caeleigh A. Landry, Michelle M. Paluszek, Thomas A. Fergus, Dean McKay, Gordon J. G. Asmundson

**Affiliations:** ^1^ Department of Psychiatry University of British Columbia Vancouver British Columbia Canada; ^2^ Department of Psychology University of Regina Regina Saskatchewan Canada; ^3^ Department of Psychology and Neuroscience Baylor University Waco Texas; ^4^ Department of Psychology Fordham University New York New York

**Keywords:** anxiety, coronavirus, COVID‐19, fear, pandemic, stress, xenophobia

## Abstract

**Background:**

Research shows that the COVID Stress Scales have a robust multifactorial structure, representing five correlated facets of COVID‐19‐related distress: (a) Fear of the dangerousness of COVID‐19, which includes fear of coming into contact with fomites potentially contaminated with SARSCoV2, (b) worry about socioeconomic costs of COVID‐19 (e.g., worry about personal finances and disruption in the supply chain), (c) xenophobic fears that foreigners are spreading SARSCoV2, (d) traumatic stress symptoms associated with direct or vicarious traumatic exposure to COVID‐19 (nightmares, intrusive thoughts, or images related to COVID‐19), and (e) COVID‐19‐related compulsive checking and reassurance seeking. These factors cohere to form a COVID stress syndrome, which we sought to further delineate in the present study.

**Methods:**

A population‐representative sample of 6,854 American and Canadian adults completed a self‐report survey comprising questions about current mental health and COVID‐19‐related experiences, distress, and coping.

**Results:**

Network analysis revealed that worry about the dangerousness of COVID‐19 is the central feature of the syndrome. Latent class analysis indicated that the syndrome is quasi‐dimensional, comprising five classes differing in syndrome severity. Sixteen percent of participants were in the most severe class and possibly needing mental health services. Syndrome severity was correlated with preexisting psychopathology and with excessive COVID‐19‐related avoidance, panic buying, and coping difficulties during self‐isolation.

**Conclusion:**

The findings provide new information about the structure and correlates of COVID stress syndrome. Further research is needed to determine whether the syndrome will abate once the pandemic has passed or whether, for some individuals, it becomes a chronic condition.

## 1 INTRODUCTION

There has been widespread emotional distress in response to the COVID‐19 pandemic. Data from China, for example, suggests that 25% of the general population have experienced moderate to severe levels of stress‐ or anxiety‐related symptoms in response to COVID‐19 (Qiu et al., 2020; Wang et al., 2020). Much remains to be learned about the sources of distress. Recent conceptualizations of COVID‐19‐related distress are narrow and unidimensional, focusing largely on fear of infection (Ahorsu et al., 2020; Lee, 2020; Mertens, Gerristen, Salemink, & Engelhard, 2020). In contrast, research and clinical observations from previous pandemics and other outbreaks suggest that the scope of distress‐related symptoms is much broader (Taylor, 2019), which led to the development of a multifactorial measure of COVID‐19‐related distress (Taylor et al., 2020). In a study of a population‐representative sample of 6,854 adults from the United States and Canada, Taylor et al. (2020) reported data on reliability (i.e., internal consistency), validity (i.e., convergent, discriminant), and factor‐analytic stability (i.e., using two independent subsamples of the full sample) of the COVID Stress Scales, which measure five inter‐correlated factors corresponding to a COVID stress syndrome. The factors are (a) DAN: Fear of the dangerousness of COVID‐19 and fear of contact with fomites (i.e., objects, surfaces) potentially contaminated with SARSCoV2, (b) SEC: Worry about the socioeconomic costs of COVID‐19 (e.g., worry about personal finances, worry about disruption in the supply chain), (c) XEN: Xenophobic fears that foreigners are spreading SARSCoV2, (d) TSS: Traumatic stress symptoms associated with direct or vicarious traumatic exposure to COVID‐19 (i.e., COVID‐19‐related nightmares, intrusive thoughts, or images), and (e) CHE: COVID‐19‐related compulsive checking and reassurance seeking.

The purpose of the present study was to extend the analysis of the data set reported on by Taylor et al. (2020) in several ways: (a) to investigate the internal structure of the syndrome via network analysis, (b) to investigate whether the syndrome is dimensional or multicategorical via latent class analysis, (c) to examine the premorbid correlates of the syndrome, (d) to investigate the concurrent correlates of the syndrome in terms of indices of current distress, and (e) to investigate how the syndrome is related to various aspects of stress and coping with self‐isolation, given that the sample was instructed by health authorities to comply with voluntary self‐isolation as part of social distancing protocols to limit the spread of infection. The overarching goal was to obtain findings that can be used to guide targeted intervention efforts for reducing distress associated with COVID‐19.

## 2 METHOD

### 2.1 Sample and data collection procedures

Data were collected from Canada and the United States using an Internet‐based self‐report survey delivered in English by Qualtrics, a commercial survey sampling, and administration company, between March 21 and April 1, 2020. As described in more detail elsewhere (Taylor et al., 2020), participation was solicited by Qualtrics using sampling of web‐panels to meet quotas based on age, gender, ethnicity, socioeconomic status, and geographic region within each country to obtain a population‐representative sample. All respondents provided informed consent. The sample comprised 6,854 adults aged 18–94 years (*M* = 49.8 years, standard deviation [*SD*] = 16.2). Almost half (47%) were female and most (57%) were employed (i.e., full‐ or part‐time, self‐employed, or on leave). Most (79%) had completed full or partial college, 18% had only completed high school or equivalent, and 3% did not graduate from high school. Most (68%) were Caucasian, with the remainder being Asian (12%), African American/Black (9%), Latino/Hispanic (6%), Native American/Indigenous (1%), or other (3%).

### 2.2 Measures

The self‐report survey comprised measures regarding demographics (e.g., age, gender, ethnicity, education, employment status), current anxiety and depression, various trait characteristics associated with psychopathology, experiences with COVID‐19 (e.g., having been diagnosed with COVID‐19, working in a job with increased risk on contact with COVID‐19), and COVID‐19‐related distress and coping.

Current anxiety and depression were assessed using the Patient Health Questionnaire‐4 (Kroenke, Spitzer, Williams, & Lowe, 2009). Trait measures pertaining to psychopathology proneness included the following: Short Health Anxiety Inventory, which measures premorbid health anxiety (Salkovskis, Rimes, Warwick, & Clark, 2002); Anxiety Sensitivity Index‐3, which measures the tendency to become anxious about arousal‐related sensations (Taylor et al., 2007); Intolerance of Uncertainty Scale‐12, which measures distress about uncertain or ambiguous situations (Carleton, Norton, & Asmundson, 2007); Perceived Vulnerability to Disease Scale (Duncan, Schaller, & Park, 2009), which measures perceived infectability and germ aversion; Disgust Propensity and Sensitivity Scale‐Revised (Fergus & Valentiner, 2009), which measures the frequency and likelihood of experiencing disgust, which is a common concomitant to fear of infection (Taylor, 2019); and the revised Obsessive‐Compulsive Inventory checking and contamination subscales (Foa et al., 2002), which measure obsessive‐compulsive checking and cleaning. To assess pre‐COVID‐19 trait characteristics, we instructed participants to respond to the trait measures as they would have before the COVID‐19 outbreak.

Worries specific to the current pandemic were assessed with the COVID Stress Scales (Taylor et al., 2020). Beliefs in COVID‐19 conspiracy theories were assessed with a 3‐item scale (e.g., “COVID‐19 is a biological weapon that got out of control”). COVID‐19‐related avoidance behaviors were assessed using two items measuring the extent of avoidance of services or places that were readily available and essential services (i.e., not closed) to participants at the time of the study: Traveling on public transport and going to grocery stores. Hygiene behaviors were assessed using eight items assessing hygiene behaviors (e.g., “Do you cover your coughs, for example, by coughing into your elbow?”). Stockpiling behaviors (panic buying) were assessed using seven items (e.g, buying of food and toiletries).

Self‐isolation stress and coping were assessed by measures of (a) stressors associated with self‐isolation (e.g., financial difficulties; 16 items), (b) aversive emotions associated with self‐isolation (e.g., anxiety, irritability; 7 items), and (c) coping strategies that might be used to make self‐isolation more tolerable (e.g., setting a routine for oneself; 28 items). Only participants who were currently in self‐isolation completed this battery. A list of items assessing aversive emotions appears in Table 1, and the list of coping strategies appears in Table 2. A list of the items assessing stressors appears in the Supporting Information. Stressors were rated with yes/no responses. Aversive emotions were rated on a 5‐point scale from 0 (*not at all*) to 4 (*extremely*). Coping strategies were assessed on a 5‐point scale ranging from 0 (*did not use this coping resource*) to 4 (*tried it and found it extremely helpful*). The rating scale for coping strategies was devised so that it was possible to assess whether or not a coping strategy was used and if it was used, its perceived efficacy in helping the respondent cope with self‐isolation. Individual items of stress and coping were examined rather than scale scores, to gain a better understanding of the nature of stressors, aversive emotions, and coping behaviors associated with self‐isolation.

**Table 1:** Correlations between the total score on the COVID Stress Scales and self‐isolation adherence, preparation, and aversive emotion variables

	*r*
Days spent in self‐isolation	.05**
Overall, how much preparation did you do for your self‐isolation?	.26***
Overall, how stressful has it been for you to be in self‐isolation?	.51***
Overall, how boring has it been for you to be in self‐isolation?	.32***
Overall, how much were you aware of your body (e.g., aches, pains, coughs, sniffles) during self‐isolation?	.34***
Overall, how anxious or worried have you been during self‐isolation?	.61***
Overall, how sad have you been during self‐isolation?	.52***
Overall, how lonely have you been during self‐isolation?	.42***
Overall, how angry or irritable have you been during self‐isolation?	.50***

*p < .01.

** *p* < .005.

*** *p* < .001.

**Table 2:** Correlations between the total score on the COVID Stress Scales and perceived efficacy of strategies for coping with self‐isolation

*r*	Respondents who tried the coping strategy (%)	Coping strategy
.02	96	Watched TV or movies
.09***	84	Kept busy cleaning or tidying up
.08***	83	Spent time talking with or texting friends on my phone
.05**	83	Reminded myself that self‐isolation is important for helping my community
.07***	78	Spent time cooking
.05	76	Spent time connecting with people via the Internet (e.g., social media)
−.05	76	Spent time on hobbies
−.04	75	Spent time reading or writing
.19***	63	Searched the Internet for news on COVID‐19
.02	63	Reminded myself that it would soon be over
.01	56	Played video games or computer games
−.05	56	Exercised (e.g., weights, sit‐ups, stationary bicycle)
.14***	56	Slept more than I normally would
.18***	51	Shopped online
.05	50	Tried new recipes
.24***	48	Ate more than I normally would
−.02	46	Set a schedule or routine for myself, such as setting specific times for meals
−.03	40	Kept busy by working at my job from home
.15***	37	Searched the Internet for new ways of keeping myself occupied (e.g., signed up for an online course or found a new hobby)
.19***	34	Monitored my symptoms (e.g., checked my temperature)
.05	29	Practiced relaxation exercises
−.01	27	Asked friends or family to deliver food or other things to my door
.00	26	Meditation
.17***	25	Consumed more alcohol or recreational drugs than I normally would
−.03	24	Yoga
.10**	23	Kept busy by trying to keep my children entertained
.20***	23	Searched for porn on the Internet
.19***	13	Met with a doctor or counselor via the Internet (e.g., phone, Skype, FaceTime)

*Note*: Correlations for perceived efficacy are shown only for respondents who actually tried the coping strategy.

*p < .01.

** *p* < .005.

*** *p* < .001.

### 2.3 Statistical procedures

#### 2.3.1 Alpha level and interpretation of correlations

Given the number of analyses reported in this article, the *α* level was set at .01 instead of .05. This corrects for inflated Type I error without unduly inflating Type II error with a more stringent correction such as a Bonferroni correction. Given the large sample size, substantively trivial correlations would be statistically significant (e.g., for *r* = .05, *p* < .001). Accordingly, to facilitate the interpretation of correlations, we used Cohen's (1988) criteria. Small, medium, and large correlations correspond to correlations of 0.10, 0.30, and 0.50, respectively. Unless stated otherwise, all analyses were conducted using the Statistical Package for the Social Sciences (SPSS, version 17.0).

### 2.4 Latent class analysis

Latent class analyses were new to the present study and were not reported in Taylor et al. (2020). To determine whether the COVID stress syndrome is a dimensional or multicategorical construct, latent class analyses were conducted using Robust Maximum Likelihood with MPlus (i.e., Maximum Likelihood using robust standard errors; Muthen & Muthen, 2017). This method was used as it is robust to departures from normality in the data distribution. The total scale score on the COVID Stress Scales was used as the input variable for the latent class analysis. Models consisting of increasing numbers of classes were evaluated until the best‐fitting model was identified, as determined by four goodness‐of‐fit indices: Akaike Information Criterion, Bayesian Information Criterion, sample‐size adjusted Bayesian Information Criterion, and bootstrap likelihood ratio test. For the first three fit indices, the best‐fitting model has the lowest value on these indices. For the bootstrap likelihood ratio test, the best‐fitting model is a model consisting of N classes, which has a significantly better (*p* < .01) fit than a model consisting of N − 1 classes, and is not significantly different from a model consisting of N + 1 classes.

#### 2.4.1 Network analysis

The rationale for network analysis is that it provides important information about relationships among elements in a network (e.g., symptoms in a syndrome). Network analysis assumes that nodes (e.g., symptoms, factors, or other psychopathological features) cluster together as they are causally linked to one another. This linking does not assume that nodes are influenced by some underlying factor; rather, network analysis assumes that nodes may directly influence one another (Epskamp, Borsboom, & Fried, 2018). If nodes causally influence one another, then changes in a central node are most likely to lead to changes in other nodes in the network through spreading of activation. Central nodes, as compared to peripheral nodes, are defining features of a network. Identifying central nodes of COVID‐19 stress can point to cardinal symptomatic features potentially conferring a direct causal effect on other features of COVID‐19‐related stress. Those cardinal features may merit particular attention when conceptualizing and dealing with COVID‐19 stress.

From the perspective of cognitive‐behavioral approaches to health anxiety, pandemics, and, trauma‐related fears (e.g., Taylor, 2017, 2019; Taylor & Asmundson, 2004), a network approach makes good theoretical sense as cognitive‐behavioral models predict that the elements in the network would interact with one another. According to cognitive‐behavioral models, negative beliefs (e.g., worry about COVID‐19 infection and its sources and consequences; DAN, SEC, and XEN) lead to checking (CHE) for information that can make the threat more predictable and controllable. CHE, in turn, exacerbates DAN as checking (e.g., checking for health‐related information on the Internet or on social media) inevitably backfires as it leads the person to encounter new, fear‐evoking information (e.g., graphic images or descriptions of sick people on the mainstream news media or shared on social media; fake news and conspiracy theories about the dangers of contagion), which in turn amplify worries (DAN, SEC, and XEN). Exposure to graphic information can also lead to traumatic stress symptoms (TSS), such as nightmares and intrusive thoughts and images. In turn, TSS can increase the perceived threat (DAN), because TSS provides vivid reminders of the dangerousness of COVID‐19.

Glasso networks (partial correlation networks) were computed using the R *qgraph* package (Epskamp, Maris, Waldorp, & Borsboom, 2016). The indices of centrality, also calculated with *qgraph*, were used to assess the nature of the connections between nodes (elements) in the network. Three indices of interconnectedness were measured (Epskamp, Cramer, Waldorp, Schmittmann, & Borsboom, 2012): Strength, betweenness, and closeness. The strength or centrality of a given node is computed as the sum of the absolute values of the weights (partial correlations) connecting that node with other nodes. A central node is one that has a large number of statistically significant (*p* < .01) links to other nodes in the network. Strength was used as the primary indicator of centrality, given that it has the most support as a stable and reliable indicator of centrality (Epskamp et al., 2018). Betweenness refers to how often a given node in the network is the most efficient (shortest) path between other nodes; that is, how important a given node is in connecting other nodes with one another. Closeness refers to how well a node is connected to other nodes in the network. Node centrality difference tests (i.e., statistical test to determine whether nodes in the network are significantly more central than other nodes) were performed using the R package *bootnet* (Epskamp et al., 2016). The stability (reliability) of the strength values for the nodes and their links was tested by the correlation of stability coefficient, also using *bootnet* (Epskamp et al., 2018).

## 3 RESULTS

### 3.1 Sample characteristics

Only 2% of the sample reported that they had been diagnosed with COVID‐19, and only 6% personally knew someone who had been infected with the coronavirus. Only 4% were healthcare workers who might come into contact with patients infected with COVID‐19. On the basis of the cutoffs for the Patient Health Questionnaire‐4 (Kroenke et al., 2009), 28% of the sample had elevated anxiety and 22% were experiencing clinically significant depressive symptoms. At the time of the study, which was in the early months of the pandemic, a total of 12% were wearing facemasks, 87% were regularly washing their hands in the prescribed manner (i.e., for at least 20 s), 59% were regularly using hand sanitizer, 95% were regularly practicing social distancing, and 48% were in self‐isolation. Data on reliability as internal consistency (coefficient *α*) for the multi‐item scales are presented in the Supporting Information.

### 3.2 Latent class analysis

The COVID Stress Scales had medium‐to‐large correlations with one another (*r*s 0.41–0.73) and, therefore, formed a coherent syndrome. Accordingly, the total sum of these scales (hereafter referred to as the total score) was used as the input variable in the latent class analysis. Fit indices for the latent class analysis (see Supporting Information) indicated that a five‐class model was the best‐fitting solution. The number (and %) of participants in each class was as follows: Class 1, *n* = 170 (3%); Class 2, *n* = 767 (11%); Class 3, *n* = 2,161 (32%); Class 4, *n* = 2,632 (38%); Class 5, *n* = 1,124 (16%). The classes ranged monotonically from low (Class 1) to high (Class 5) in terms of the scores on the total score on the COVID Stress Scales.

To further characterize the nature of the classes, respondents were classified in terms of the global severity of their symptoms on the Patient Health Questionnaire‐4 (i.e., anxiety and depression), using the cutoffs described by Kroenke et al. (2009). On the basis of the Patient Health Questionnaire‐4, respondents were classified as having little or no distress, mild distress, moderate distress, or severe distress. In Classes 1–2, most (>90%) of respondents reported no more than mild distress. In Class 4, 46% reported mild‐moderate distress, and 10% reported severe distress. In Class 5, most respondents (59%) reported severe distress (see Supporting Information for further details).

If the latent class cutoffs were to be used for diagnostic purposes, the results suggest that 16% of the sample (class 5) had severe COVID stress syndrome. The five classes differed quantitatively rather than qualitatively in the severity of scores on each of the COVID Stress Scales (see Supporting Information). Accordingly, in the following analyses, the COVID stress syndrome is treated as a quasi‐dimensional construct, assessed by total score.

### 3.3 Network analysis

**Figure 1:**
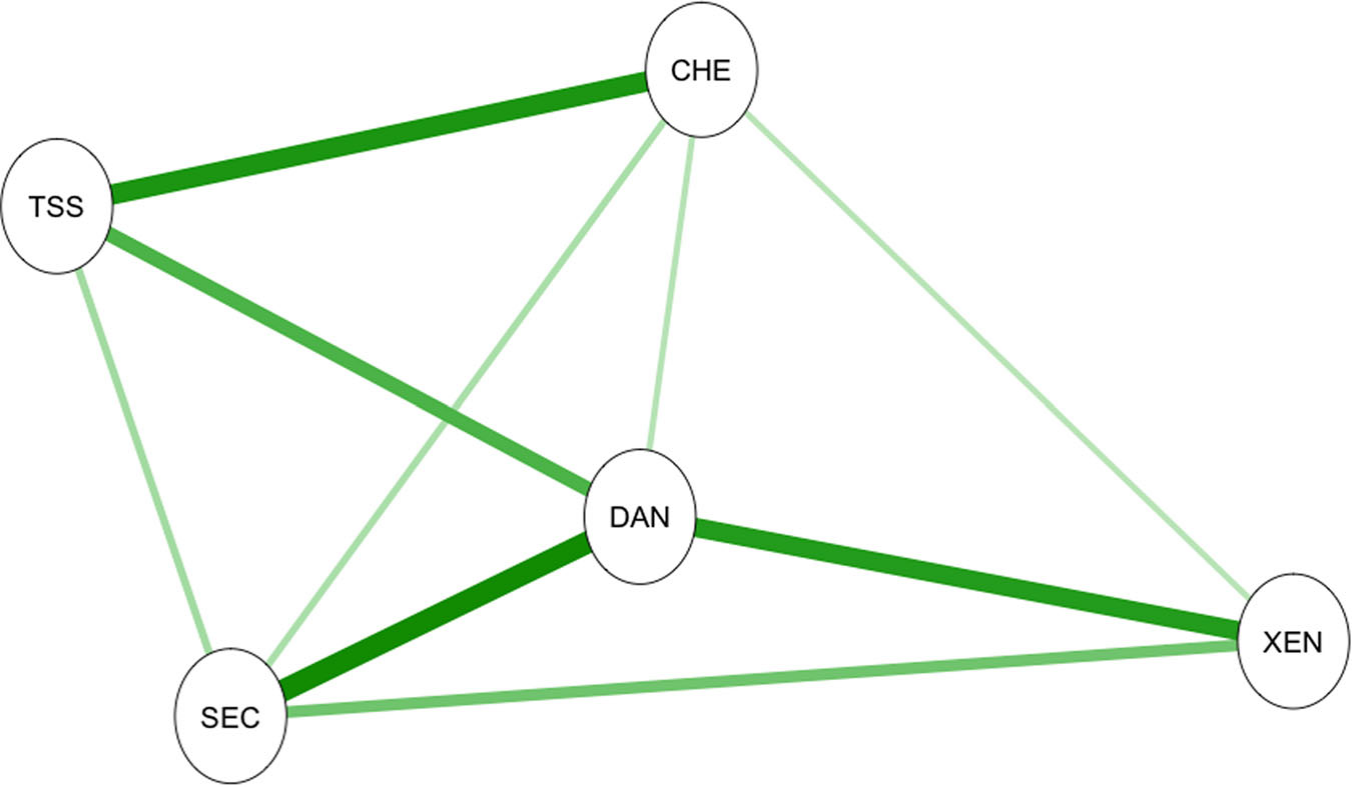
Network analysis: Strength of interconnections (partial correlations) among the elements of the COVID stress syndrome. Stronger connections are indicated by shorter and thicker lines. Only significant (*p* < .01) connections are depicted. CHE, COVID‐19‐related compulsive checking and reassurance seeking; DAN, COVID‐19‐related danger and contamination fears; SEC, fears of COVID‐19‐related socioeconomic consequences; TSS, COVID‐19‐related traumatic stress symptoms; XEN, COVID‐19‐related xenophobia

**Figure 2:**
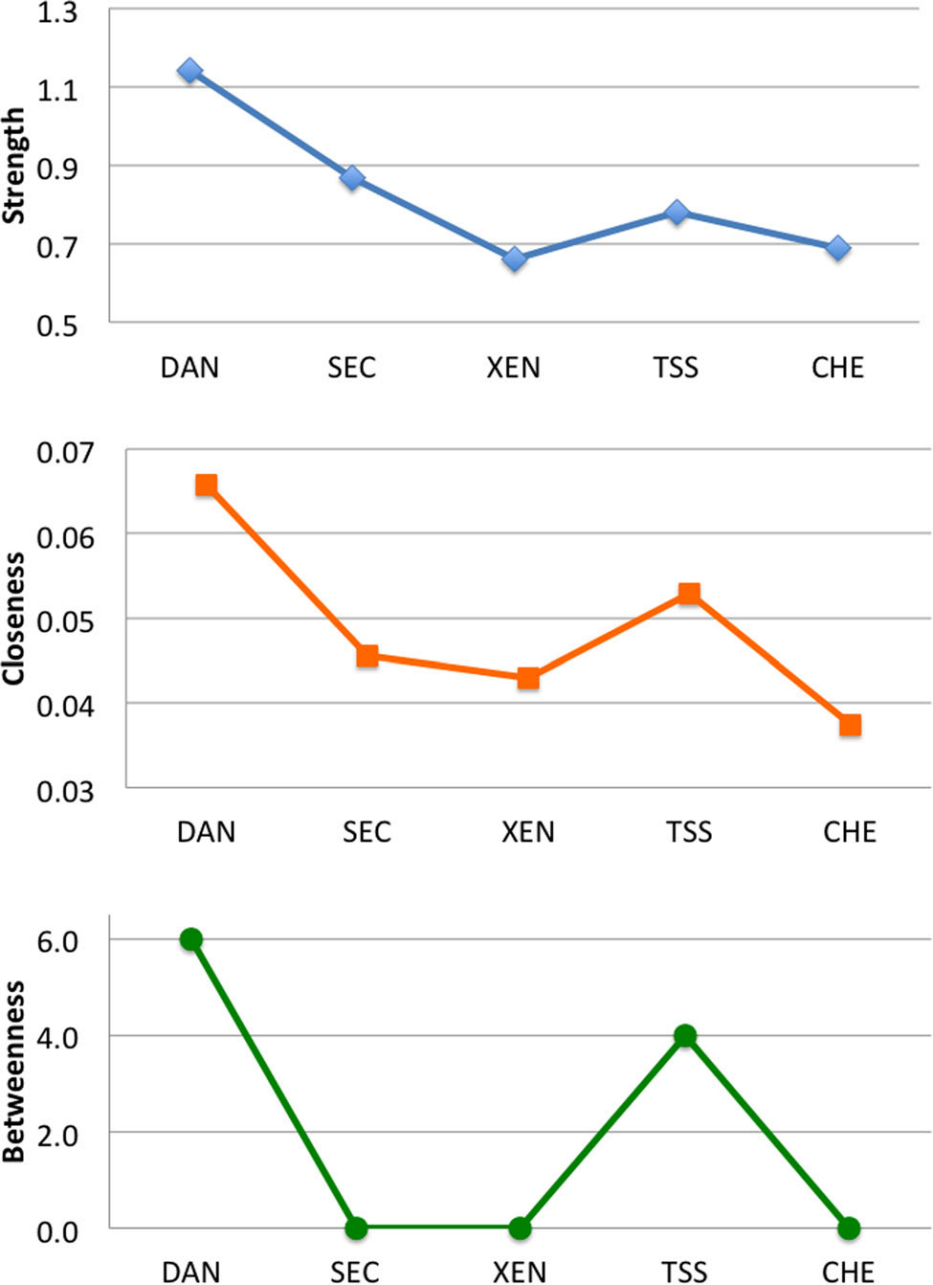
Centrality indices for network analysis. Large numbers indicate that a given element had greater importance in the network, as indicated by its connections with other elements in the network. Results show that DAN was the central element in the network. CHE, COVID‐19‐related compulsive checking and reassurance seeking; DAN, COVID‐19‐related danger and contamination fears; SEC, fears of COVID‐19‐related socioeconomic consequences; TSS, COVID‐19‐related traumatic stress symptoms; XEN, COVID‐19‐related xenophobia

Figure 1 shows the significant (*p* < .01) links (partial correlations) between nodes in the network defined by the COVID Stress Scales. The Correlation of Stability coefficients were 0.75 for both the nodes and their links (pathways). These values exceed the cutoff of 0.50 (Epskamp et al., 2018), suggesting that the values of the nodes and their links were stable (reliable). In Figure 1, the strength of the connection between nodes is indicated by shorter, thicker lines. Figure 2 presents the graphs of values for strength, betweenness, and closeness. The figures, taken together, indicate that DAN is central to the network, with particularly strong links to SEC and XEN. The strength values of the nodes were as follows: DAN = 1.14, SEC = 0.87, XEN = 0.66, TSS = 0.78, and CHE = 0.69. Pairwise comparisons using *bootnet* indicated that all pairs of nodes significantly differed from one another in their strengths (*p*s < .01), except for the strengths of XEN and CHE, which were not significantly different from one another. Thus, the central node in the network was DAN, followed by SEC. The most peripheral nodes in the network were XEN and CHE. Although DAN was the central node, it was more strongly linked to some nodes than others. DAN had significantly stronger links to SEC, XEN, and TSS, compared with its link to CHE (*p* < .01).

Network analysis also revealed a strong link between TSS and CHE; that is, COVID‐19‐related reexperiencing symptoms (e.g., intrusive thoughts or images and nightmares related to COVID‐19) were strongly linked to COVID‐19‐related checking of news media and reassurance seeking about one's health from friends, loved ones, or health professionals. This finding may reflect a dose–effect whereby a greater degree of exposure to COVID‐19‐related news or social media (via checking) leads to a greater frequency of unwanted, intrusive thoughts, images, or nightmares about COVID‐19.

### 3.4 Correlates of the COVID stress syndrome

#### 3.4.1 Relationship to demographic features

The samples from the United States and Canada did not differ in total scores on the COVID Stress Scales (*t* = 2.36, *df* = 6,852, *p* > .01). Contrary to the stereotype that younger people are relatively unconcerned about COVID‐19, results indicated that age and the total score on the COVID Stress Scales had a small‐to‐medium *negative* correlation (*r* = −.25, *p* < .001). Income level had a small but statistically significant negative correlation with the total score (*r* = −.05, *p* < .001). Women tended to have higher total scores than men (*t* = 12.64, *df* = 6,845, *p* < .001), as did people who were unemployed (*t* = 3.12, *df* = 6,852, *p* = .002) and people who were less educated (i.e., did not have a college education (*t* = 4.52, *df* = 6,852, *p* < .001). Means and *SD*s for these analyses and those presented below appear in the Supporting Information materials.

In terms of ethnicity, four main groups were compared: Asian, Black/African American, Hispanic, and Caucasian. The groups significantly differed on the total score; *F*(3, 6,528) = 60.86, *p* < .001. Post hoc Student–Neuman–Keuls tests (*α* = .01) indicated that Caucasians tended to have the lowest scores, people of Black/African American ancestry had intermediate scores, and people of Asian and Hispanic ancestry had highest scores (see Supporting Information for means and *SD*s).

People who reported that they had been diagnosed with COVID‐19 had significantly higher total scores on the COVID Stress Scales than people who had not been diagnosed (*t* = 16.13, *df* = 6,852, *p* < .001). As noted above, only 2% of the sample had been diagnosed with COVID‐19 and the results reported in this article did not change when those 2% were removed from the analyses. Regarding occupational status, people who were healthcare workers did not differ from other individuals in their total scores (*t* = 0.81, *df* = 6,847, *p* > .40). Similarly, people in occupations that put them at increased risk of contracting COVID‐19 (e.g., grocery store workers) did not differ from other individuals on the total score (*t* = 1.20, *df* = 6,848, *p* > .20).

#### 3.4.2 Premorbid correlates of the COVID stress syndrome

The presence (vs. absence) of preexisting general medical conditions (e.g., diabetes) were not associated with heightened total scores (*t* = 0.58, *df* = 6,846, *p* > .50). However, people with a preexisting (past year) mental health disorder had significantly higher total scores (*t* = 11.70, *df* = 6,845, *p* < .001). People with high scores on the total score were also more likely (*p* < .001) to have higher and generally large correlations with premorbid health anxiety (*r* = .49), anxiety sensitivity (*r* = .59), intolerance of uncertainty (*r* = .54), disgust propensity (*r* = .44), disgust sensitivity (*r* = .52), perceived infectability (*r* = .35), germ aversion (*r* = .37), and obsessive‐compulsive contamination concerns (*r* = .53) and checking rituals (*r* = .50).

#### 3.4.3 Concurrent features of the COVID stress syndrome

People with high scores on the total score on the COVID Stress Scales were more likely (*p* < .001) to be concurrently anxious (*r* = .54) and depressed (*r* = .49), and to believe in COVID‐19 conspiracy theories (*r* = .37), perform hygiene behaviors (*r* = .33), stockpile food and others supplies (*r* = .37), and avoid public transport (*r* = .30) and grocery stores (*r* = .45) for fear of getting COVID‐19. The correlations were medium to large in size. People with high total scores were also most likely to wear facemasks (*t* = 23.67, *df* = 6,841, *p* < .001).

#### 3.4.4 Stressors associated with self‐Isolation

A total of 3,304 participants were in voluntary self‐isolation at the time of assessment, for a mean of 10 days (*SD* = 8 days). Note that US states and Canadian provinces and territories differed in their recommendations for self‐isolation at the time of the study; some recommended self‐isolation, whereas others did not. Accordingly, only a portion of our participants were in voluntary self‐isolation at the time of the study.

Table 1 shows that the total score on the COVID Stress Scales had a statistically significant but substantively small correlation with the number of days in self‐isolation. Table 1 further shows that the total score had significant and generally large correlations with indices of distress experienced during self‐isolation; that is, people with a more severe COVID stress syndrome found self‐isolation to be highly distressing, assessed in terms of a range of negative emotions. Similarly, people with high total scores were more likely (*p* < .001) to report problems with a range of stressors during self‐isolation, including running low on prescription medicines, fights or arguments, difficulty taking care of family members or pets, financial problems, lack of personal space, and crowded living conditions (see Supporting Information for details). People who lived alone, compared to those who lived with one or more other people, tended to have lower total scores (*t* = 4.88, *df* = 3,302, *p* < .001).

#### 3.4.5 Coping with self‐Isolation

Of the 28 coping strategies assessed, people with high scores on the total score on the COVID Stress Scales were more likely to have tried all the coping strategies (*p*s < .001), including adaptive coping strategies (e.g., setting a routine for oneself, spending time connecting with people via the Internet or text messaging) and maladaptive coping strategies (e.g., over‐eating, consuming excessive drugs or alcohol; see the Supporting Information for details). Thus, people with COVID stress syndrome were not passive. They were actively trying to find ways of making self‐isolation more tolerable. Table 2 shows the frequency of use of coping strategies along with correlations between total scores and the perceived efficacy of coping strategies. The table shows that the most commonly used strategies involved watching TV or movies, cleaning or tidying up, maintaining social contact with friends or family, and reminding oneself that self‐isolation was important in helping the community. The least frequently used coping strategy involved accessing medical or mental health services. The perceived efficacy of coping strategies was largely unrelated to the total score on the COVID Stress Scales. The statistically significant correlations were all small or small‐to‐medium in magnitude. People with high total scores were more likely to engage in emotion‐focused coping (e.g., over‐eating) and more likely to meet with a doctor or counselor.

## 4 DISCUSSION

Our findings suggest that the psychological footprint of COVID‐19 is likely to be more substantial than the medical footprint. That is, at the time of conducting this study the number of people emotionally affected by COVID‐19 far exceeded the number of people who had been infected. Only 2% reported that they had been diagnosed with COVID‐19, and only 6% were personally acquainted with someone who had COVID‐19. And yet, the latent class analysis indicated that 38% experienced some degree of distress (Class 4) and an additional 16% were highly distressed (Class 5) and likely in need of mental health services. Worry about infection and its consequences can contribute to behaviors such as panic buying, which can further exacerbate emotional distress and social disruption (Taylor, 2019). Despite widespread distress, the least commonly used coping strategies among those assessed in the study were to seek out medical or mental health services. This raises the question of whether respondents were sufficiently aware of, or had accessibility to, mental health services available via telephone or the Internet. Respondents were more likely to resort to over‐eating or excessive use of drugs or alcohol in an attempt to cope with distress associated with self‐isolation. Respondents tended to regard self‐defeating coping strategies such as over‐eating and over‐using drugs and alcohol as efficacious in coping with self‐isolation (Table 2). Such strategies may be effective in alleviating distress in the short term but can lead to longer‐term problems.

Recent conceptualizations of COVID‐19‐related distress tend to be narrow and unidimensional, focusing largely on fear of infection (Ahorsu et al., 2020; Lee, 2020; Mertens et al., 2020). In contrast, research based on the COVID Stress Scales suggests a broader, more nuanced conceptualization. Research from the present study and our previous investigation (Taylor et al., 2020) provides evidence of a COVID stress syndrome characterized by a network of interconnected symptoms, with fear of the dangerousness of COVID‐19 at the core, interconnecting to socioeconomic concerns, xenophobia, traumatic stress symptoms, and compulsive checking and reassurance seeking. The network, in turn, is associated with other variables such as panic buying, excessive avoidance, and high levels of distress and maladaptive coping during self‐isolation.

The causal status of the links in the network remains to be further investigated. Cognitive‐behavioral models posit that the elements in the network influence one another, as described above. If this is the case, then given that worry about the dangerousness of COVID‐19 is central, changing this worry (e.g., via cognitive‐behavioral therapy) would lead to changes in all other variables in the network. This suggests, for example, that societally disruptive phenomena such as COVID‐19‐related xenophobia (including racial discrimination) could be reduced by reducing worries about becoming infected with COVID‐19. Such findings have potentially important implications for planning mental health services (for further discussion of service planning, see Vigo et al., 2020).

The present study has three main limitations to be addressed in future research. The manner in which COVID stress syndrome fits in with DSM‐5 or ICD‐11 disorders was not assessed. In some cases, the COVID stress syndrome could be a disorder in its own right, whereas in other cases it might be a part of some other disorder, such as generalized anxiety disorder, obsessive compulsive disorder, illness anxiety disorder, or posttraumatic stress disorder. Indeed, COVID stress syndrome has features of all of these disorders. Second, the stability of the syndrome needs to be assessed in prospective studies. It could be a short‐lived adjustment‐related problem, abating once the COVID‐19 pandemic subsides, or it could become, in some cases, a chronic condition. Third, functional impairment was not assessed, which is important in determining the severity and impact of a clinical condition, and whether it should be regarded as a mental disorder. Functional impairment was difficult to assess, given that people were requested by health authorities to voluntarily self‐isolate and therefore, in many cases, unable to perform their usual social and occupational activities.

## 5 CONCLUSION

Despite the above‐mentioned limitations, the present study offers several novel findings and demonstrates that COVID‐19‐related stress reactions are more complex than a simple unidimensional fear of infection. The COVID stress syndrome is a complex phenomenon involving various types of fears, checking and reassurance seeking, and reexperiencing symptoms, along with associated features such as excessive avoidance and panic buying. Understanding these reactions and their inter‐relations can inform the development of targeted interventions to reduce COVID‐19‐related distress.

## CONFLICT OF INTERESTS

Dr. Taylor receives financial support through royalties from various book publishers and from editorial duties as Associate Editor of the Journal of Obsessive Compulsive and Related Disorders. Dr. Asmundson is the Editor‐in‐Chief of the Journal of Anxiety Disorders and Development Editor of Clinical Psychology Review. He receives financial support through payments for his editorial work on the aforementioned journals and royalties from various book publishers. Dr. McKay is Associate Editor of Behavior Therapy, Journal of Obsessive Compulsive and Related Disorders, and the Bulletin of the Menninger Clinic. He receives royalties through his editorial work on the aforementioned journals, as well as from various book publishers. Dr. McKay also receives funding from a private investment company for the development of technology assisted methods for the treatment of fear of public speaking.

## ETHICS STATEMENT

The research described in this article was approved by the Research Ethics Board of the University of Regina (REB# 2020‐043).

## Supporting information

Supporting information

## Data Availability

Data available on request from the authors.
