# Supplementary material for: COVID stress syndrome: Concept, structure, and correlates
Source: Depress Anxiety. 2020 Jul 5;37(8):706–14. doi: 10.1002/da.23071 (PMC7362150; doi:10.1002/da.23071)
Supplement: Supplementary file 1 — Supporting information [file DA-37-706-s001.pdf]

CSS Supplement

Network Analyses

	Strength	Closeness	Betweenness
DAN	1.142	0.066	6.000
SEC	0.886	0.046	0.000
XEN	0.661	0.043	0.000
TSS	0.780	0.053	4.000
CHE	0.690	0.038	0.000

99%CIs. Significant = CIs that don't overlap with zero. Bold = significantly different strengths

Difftest	Strength
DAN vs SEC	**-0.346 to -0.210**
DAN vs XEN	**-0.540 to -0.417**
DAN vs TSS	**-0.422 to -0.295**
DAN vs CHE	**-0.506 to -0.387**
SEC vs XEN	**-0.265 to -0.153**
SEC vs TSS	**-0.147 to -0.031**
SEC vs CHE	**-0.238 to -0.125**
XEN vs TSS	**0.065 to 0.171**
XEN vs CHE	-0.026 to 0.076
TSS vs CHE	**-0.149 to -0.034**

Fit indices for the latent class analyses

Classification variable: Total score on the COVID Stress Scales

Number of classes	AIC	BIC	Adj. BIC	BLRT:Chi-sq	BLRT:df	BLRT:p
1	65540.91	65554.57	65548.22	--	--	--
2	64241.02	64275.18	64259.29	1305.89	3	< .001
3	63998.80	64053.46	64028.04	248.22	3	< .001
4	63865.60	63940.76	63905.80	139.21	3	< .001
5	**63803.73**	**63899.39**	**63854.90**	**67.87**	**3**	**< .001**
6	63808.65	63924.79	63870.79	1.11	3	> .10
7	63815.36	63952.01	63888.45	0.00	3	> .10

Best-fitting model = 5 classes

Class	I		II		III		IV		V		F(df=4, 6849)	p	SNK (alpha=.01)
N	170		767		2161		2632		1124				
%	2.5		11.2		31.5		38.4		16.4				
Scores	M	SD	M	SD	M	SD	M	SD	M	SD			
DANCON	0.4	0.7	3.7	2.6	10.9	4.3	21.5	6.7	35.9	7.5	5379.29	< .001	I < II < III < IV < V
SEC	0.2	0.4	1.1	1.5	3.7	3.0	8.7	4.6	16.4	5.1	2673.90	< .001	I < II < III < IV < V
XEN	0.1	0.4	0.8	1.4	2.8	3.0	7.1	5.2	15.0	6.0	1864.90	< .001	I < II < III < IV < V
TSS	0.1	0.2	0.2	0.7	1.2	1.9	4.4	4.1	11.6	6.2	1728.83	< .001	I, II < III < IV < V
CHE	0.2	0.5	2.1	2.3	3.9	3.2	7.3	4.3	13.3	5.7	1383.60	< .001	I < II < III < IV < V
CSSTOT	0.9	0.9	7.9	2.8	22.5	5.6	49.0	10.7	92.2	18.0	11475.25	< .001	I < II < III < IV < V

Cross-tabulation of latent classes with PHQ-4 classifications

PHQ-4 total scores	Interpretation
0-2	normal
3-5	mild
6-8	moderate
9-12	severe

	N					%				%	
Class	normal	mild	moderate	severe	tot	normal	mild	moderate	severe	normal or mild	moderate or severe
1	149	11	7	3	170	88	6	4	2	94	6
2	674	72	8	13	767	88	9	1	2	97	3
3	1562	418	133	48	2161	72	19	6	2	92	8
4	1137	775	460	260	2632	43	29	17	10	73	27
5	191	274	300	359	1124	17	24	27	32	41	59

Scores on CSSTOT

	M	SD
Asian	50.2	29.7
Black	46.6	31.7
Hispanic	51.5	32.4
Caucasian	39.0	27.3

Comparisons on CSStot

$F(3,6528) = 60.86$, $p < .001$

SNK ($p<.01$):

Caucasian < Black, Asian, Hispanic

Black < Hispanic

Black = Asian

Hispanic = Asian

Group comparisons: DV= CSSTOT

	Female		Male				
	M	SD	M	SD	t	df	p
Female gender	46.6	29.5	37.8	27.7	12.64	6845	< .001
	No		Yes				
College	M	SD	M	SD	t	df	p
	45.0	30.5	41.1	28.3	4.52	6852	< .001
	No		Yes				
Unemployed	M	SD	M	SD	t	df	p
	41.6	28.8	45.2	29.0	3.12	6852	0.002
	No		Yes				
Diagnosis of COVID-19	M	SD	M	SD	t	df	p
	41.1	28.1	80.0	36.0	16.13	6852	< .001
	Cda		US				
Country	M	SD	M	SD	t	df	p
	41.1	27.5	42.8	30.1	2.36	6852	0.018
	No		Yes				
Preexisting medical condition	M	SD	M	SD	t	df	p
	41.7	28.6	42.2	29.1	0.58	6846	0.563
	No		Yes				
Preexisting (past year) mental health condition	M	SD	M	SD	t	df	p
	40.0	28.0	50.4	30.8	11.7	6845	< .001
	No		Yes				
Healthcare worker who might come in contact	M	SD	M	SD	t	df	p
	41.9	28.7	43.3	31.7	0.81	6847	0.418
	No		Yes				
Other occupation of incr risk	M	SD	M	SD	t	df	p
	41.8	29.0	42.9	28.2	1.2	6848	0.231

	No		Yes				
Wear a facemask because of COVID concerns	M	SD	M	SD	t	df	p
	39.0	27.0	63.5	32.6	23.67	6841	< .001

Stressors encountered during self-isolation: DV = CSSTOT

No		Yes					
M	SD	M	SD	t	df	p	
41.9	27.2	58.1	33.2	13.44	3300	< .001	Running low on food
42.1	27.2	64.0	34.1	15.88	3298	< .001	Running low on cleaning supplies
42.1	27.2	64.0	34.1	15.88	3298	< .001	Running low on prescription medicines
42.1	27.4	61.6	33.0	14.87	3300	< .001	Running low on toiletries
44.2	28.4	78.0	36.4	12.54	3298	< .001	Difficulty taking care of a pet or pets
42.5	27.7	58.7	32.9	12.47	3300	< .001	Fights or arguments with people
40.6	26.9	56.8	31.7	14.97	3300	< .001	Financial problems
41.9	28.0	53.7	30.8	10.71	3297	< .001	Running out of things to do
42.2	27.8	60.7	32.1	14.05	3299	< .001	Not having enough physical space to move around in
43.3	28.1	59.3	33.6	10.77	3295	< .001	Not having enough personal space
44.4	28.6	66.5	37.9	8.92	3296	< .001	Difficulty taking care of children
44.7	28.8	64.1	36.8	7.52	3301	< .001	Difficulty taking care of an elderly loved one
46.7	29.7	40.5	27.5	4.88	3302	< .001	Lives alone

Coping with self-isolation: DV = CSSTOT

No		Yes						
M	SD	M	SD	t	df	p		
40.0	26.5	51.8	31.3	11.00	3302	< .001	tried it; yes/no	Set a schedule or routine for myself, such as setting specific times for meals
35.4	25.6	48.5	29.8	11.08	3302	< .001	tried it; yes/no	Spent time connecting with people via the internet (e.g., social media)
41.3	27.0	56.4	32.6	13.49	3302	< .001	tried it; yes/no	Asked friends or family to deliver food or other things to my door
42.8	27.8	46.3	30.0	2.94	3302	0.003	tried it; yes/no	Spent time reading or writing
40.1	26.6	47.1	30.1	5.93	3302	< .001	tried it; yes/no	Spent time on hobbies
37.0	29.7	45.7	29.3	3.23	3302	0.001	tried it; yes/no	Watched TV or movies
40.8	25.8	49.0	31.4	8.01	3302	< .001	tried it; yes/no	Played video games or computer games
37.9	25.9	47.5	30.0	7.87	3302	< .001	tried it; yes/no	Spent time cooking
40.0	26.2	50.7	31.4	10.68	3302	< .001	tried it; yes/no	Tried new recipes
32.9	24.1	52.8	29.7	19.83	3302	< .001	tried it; yes/no	Searched the Internet for news on COVID-19
38.2	25.0	57.9	32.2	19.62	3302	< .001	tried it; yes/no	Searched the Internet for new ways of keeping myself occupied (e.g., signed up for an online course or found a new hobby)
36.1	27.8	47.3	29.4	8.19	3302	< .001	tried it; yes/no	Spent time talking with or texting friends on my phone
34.3	24.3	47.6	29.8	9.75	3302	< .001	tried it; yes/no	Kept busy cleaning or tidying up
41.2	26.6	51.9	32.2	10.45	3302	< .001	tried it; yes/no	Kept busy by working at my job from home
41.1	26.6	60.0	33.5	16.14	3302	< .001	tried it; yes/no	Kept busy by trying to keep my children entertained
41.5	27.3	48.4	30.6	6.75	3302	< .001	tried it; yes/no	Exercised (e.g., weights, sit-ups, stationary bicycle)
41.5	26.9	58.0	33.1	14.32	3302	< .001	tried it; yes/no	Yoga
41.1	26.4	57.8	33.6	14.79	3302	< .001	tried it; yes/no	Meditation
40.6	26.6	57.4	32.4	15.31	3302	< .001	tried it; yes/no	Practiced relaxation exercises
36.7	24.9	50.6	30.6	13.44	3302	< .001	tried it; yes/no	Reminded myself that it would soon be over

33.9	25.7	47.8	29.6	10.31	3302	< .001	tried it; yes/no	Reminded myself that self-isolation is important for helping my community
36.7	25.1	54.9	30.8	18.74	3302	< .001	tried it; yes/no	Ate more than I normally would
41.1	27.0	58.2	32.4	15.11	3302	< .001	tried it; yes/no	Consumed more alcohol or recreational drugs than I normally would
37.4	25.5	51.7	30.7	14.30	3302	< .001	tried it; yes/no	Slept more than I normally would
42.2	27.3	56.5	33.3	11.95	3302	< .001	tried it; yes/no	Searched for porn on the internet
38.9	26.2	51.7	30.9	12.76	3302	< .001	tried it; yes/no	Shopped online
37.3	24.8	61.3	31.3	24.00	3302	< .001	tried it; yes/no	Monitored my symptoms (e.g., checked my temperature)
42.2	27.2	66.8	34.1	17.05	3302	< .001	tried it; yes/no	Met with a doctor or counsellor via the internet (e.g., phone, Skype, FaceTime)

Correlation with CSSTOT (positive r = more effective coping strategy)

r	no. of respondents who used the strategy	% of respondents who tried the coping strategy	**If a coping strategy was tried, rating of its effectiveness**
.02	3,181	96	Watched TV or movies
.09***	2,760	84	Kept busy cleaning or tidying up
.08***	2,754	83	Spent time talking with or texting friends on my phone
.05**	2,746	83	Reminded myself that self-isolation is important for helping my community
.07***	2,573	78	Spent time cooking
.05	2,526	76	Spent time connecting with people via the internet (e.g., social media)
-.05	2,495	76	Spent time on hobbies
-.04	2,467	75	Spent time reading or writing
.19***	2,080	63	Searched the Internet for news on COVID-19
.02	2,076	63	Reminded myself that it would soon be over
.01	1,865	56	Played video games or computer games
-.05	1,861	56	Exercised (e.g., weights, sit-ups, stationary bicycle)
.14***	1,856	56	Slept more than I normally would
.18***	1,680	51	Shopped online
.05	1,665	50	Tried new recipes
.24***	1,581	48	Ate more than I normally would
-.02	1,521	46	Set a schedule or routine for myself, such as setting specific times for meals
-.03	1,308	40	Kept busy by working at my job from home
.15***	1,217	37	Searched the Internet for new ways of keeping myself occupied (e.g., signed up for an online course or found a new hobby)
.19***	1,116	34	Monitored my symptoms (e.g., checked my temperature)
.05	945	29	Practiced relaxation exercises
-.01	900	27	Asked friends or family to deliver food or other things to my door
.00	858	26	Meditation
.17***	841	25	Consumed more alcohol or recreational drugs than I normally would
-.03	788	24	Yoga
.10**	754	23	Kept busy by trying to keep my children entertained
.20***	749	23	Searched for porn on the internet
.19***	435	13	Met with a doctor or counsellor via the internet (e.g., phone, Skype, FaceTime)

Coefficient alpha for multi-item scales

Scale	alpha	M	SD
Patient Health Questionnaire-4	0.90	3.2	3.4
Short Health Anxiety Inventory	0.90	10.9	6.7
Anxiety Sensitivity Index-3	0.94	18.0	14.3
Intolerance of Uncertainty Scale-12	0.91	30.6	9.9
Perceived Vulnerability to Disease Scale-Perceived infectibility	0.86	23.8	8.1
Perceived Vulnerability to Disease Scale-Germ avoidance	0.73	35.8	8.4
Disgust Propensity Scale-Revised	0.82	16.1	4.1
Disgust Sensitivity Scale-Revised	0.79	12.9	4.7
Obsessive-Compulsive Inventory, Revised, Contamination Subscale	0.86	3.3	3.2
Obsessive-Compulsive Inventory, Revised, Checking Subscale	0.86	3.4	3.0
COVID Stress Scales - DAN	0.95	18.0	11.7
COVID Stress Scales - SEC	0.91	7.3	6.3
COVID Stress Scales - XEN	0.93	6.2	6.3
COVID Stress Scales - TSS	0.93	4.0	5.3
COVID Stress Scales - CHE	0.85	6.5	5.4
Beliefs in COVID-19 conspiracy theories	0.85	4.6	3.3
Hygiene behaviors	0.54	5.7	0.2
Stockpiling behaviors	0.83	1.3	0.3
